# Degradation of Fructans and Production of Propionic Acid by *Bacteroides thetaiotaomicron* are Enhanced by the Shortage of Amino Acids

**DOI:** 10.3389/fnut.2014.00021

**Published:** 2014-12-05

**Authors:** Signe Adamberg, Katrin Tomson, Heiki Vija, Marju Puurand, Natalja Kabanova, Triinu Visnapuu, Eerik Jõgi, Tiina Alamäe, Kaarel Adamberg

**Affiliations:** ^1^Tallinn University of Technology, Tallinn, Estonia; ^2^Competence Center of Food and Fermentation Technologies, Tallinn, Estonia; ^3^National Institute of Chemical Physics and Biophysics, Tallinn, Estonia; ^4^Department of Genetics, Institute of Molecular and Cell Biology, University of Tartu, Tartu, Estonia

**Keywords:** *Bacteroides thetaiotaomicron*, fructan, levan, FOS, defined medium, isothermal microcalorimetry, amino acid metabolism, metabolic flux analysis

## Abstract

*Bacteroides thetaiotaomicron* is commonly found in the human colon and stabilizes its ecosystem by catabolism of various polysaccharides. A model of cross-talk between the metabolism of amino acids and fructans in *B. thetaiotaomicron* was proposed. The growth of *B. thetaiotaomicron* DSM 2079 in two defined media containing mineral salts and vitamins, and supplemented with either 20 or 2 amino acids, was studied in an isothermal microcalorimeter. The polyfructans inulin (from chicory) and levan (synthesized using levansucrase from *Pseudomonas syringae*), two fructooligosaccharide preparations with different composition, sucrose and fructose were tested as substrates. The calorimetric power-time curves were substrate specific and typically multiauxic. A surplus of amino acids reduced the consumption of longer oligosaccharides (degree of polymerization > 3). Bacterial growth was not detected either in the carbohydrate free medium containing amino acids or in the medium with inulin as a sole carbohydrate. In amino acid-restricted medium, fermentation leading to acetic acid formation was dominant at the beginning of growth (up to 24 h), followed by increased lactic acid production, and mainly propionic and succinic acids were produced at the end of fermentation. In the medium supplemented with 20 amino acids, the highest production of d-lactate (82 ± 33 mmol/gDW) occurred in parallel with extensive consumption (up to 17 mmol/gDW) of amino acids, especially Ser, Thr, and Asp. The production of Ala and Glu was observed at growth on all substrates, and the production was enhanced under amino acid deficiency. The study revealed the influence of amino acids on fructan metabolism in *B. thetaiotaomicron* and showed that defined growth media are invaluable in elucidating quantitative metabolic profiles of the bacteria. Levan was shown to act as an easily degradable substrate for *B. thetaiotaomicron*. The effect of levan on balancing or modifying colon microbiota will be studied in further experiments.

## Introduction

In last decades, the genetic composition and species diversity of the gastrointestinal tract ecosystem have been studied intensively, but the dynamics of the microbiota at the metabolic level are still insufficiently understood. More than 90% of 16S rRNA gene sequences of the human intestinal microflora belong to the phyla *Firmicutes* and *Bacteroidetes* (1), with the genus *Bacteroides* accounting for 14–40% of cultivable bacteria in human feces (2). The colon microbiota members, particularly *Bacteroides* bacteria, are highly efficient degraders of complex polysaccharides, including mucins and dietary fibers (3). Dietary fiber compounds are resistant to human digestive enzymes and reach the colon virtually unmodified (4). As a result, the content and composition of polysaccharides in the diet shape the colon microbiota and certain types of food fibers can be used as prebiotics to support the host’s health (5, 6).

Inulin, a β 2,1 linked fructan [degree of polymerization (DP) 2–60], and inulin-derived fructooligosaccharides (FOS_inu_, DP < 10) are abundant and the best studied dietary fibers (prebiotic compounds). For example, 100 g of wheat bread contain 0.5–2 and 100 g of banana 0.3–0.7 g of inulin (7). The ability of inulin to promote the growth of beneficial gut bacteria is well documented (4). The prebiotic effects of levan (a β 2,6 linked fructan) and levan-type oligosaccharides (FOS_lev_) have been much less studied. Nevertheless, enhanced bifidogenic properties and the chemical stability of FOS_lev_ and neo-FOS (e.g., neokestose), compared to FOS_inu_, have been reported (8, 9). Levan and respective FOS are produced by extracellular enzymes of several bacteria, e.g., bacilli, lactobacilli, and proteobacteria (10–13), or synthesized in several plants (14–16). Here, it should be noted that, quite recently, levan was shown to be a specific carbon source for *B. thetaiotaomicron* strains. This species possesses a complete polysaccharide utilizing locus (PUL) encoding not only β-fructofuranosidases but also an endo-levanase (17). Earlier, Salyers et al. (18) reported considerable variation in the usage of different polysaccharides from plants and mucins by several *Bacteroides* species, including those isolated from the human colon. Yet, little is known about metabolic activities and product profiles of *B. thetaiotaomicron* growing on fructans. In particular, quantitative data on the amino acid metabolism of *Bacteroides* in the presence of complex carbohydrates, such as polyfructans, are lacking. Varel and Bryant (19) reported that *B. fragilis* and *B. thetaiotaomicron* were able to synthesize all necessary amino acids from inorganic ammonia when grown in a defined minimal medium. In contrast, Arg, Asp, Gly, His, h-Pro, Leu, Ile, Lys, Ser, Thr, Trp, Tyr, and Val were shown to be essential for growth of *Bacteroides* strains from different species in a defined medium containing glucose (20). The genomic data and *in silico* models of *B. thetaiotaomicron* suggest several proteins for the use and synthesis of amino acids and the production of branched-chain fatty acids (BCFA) (21, 22), yet, experimental data on this subject are scarce. *B. thetaiotaomicron* contributes to stability and diversity in the colon ecosystem by the catabolism of a wide variety of polysaccharides, thus acting as a primary degrader and fermenter (23–26). It is also hypothesized that *B. thetaiotaomicron* synergistically enhances butyrate formation in the colon by providing acetate for butyrate producers (e.g., *Eubacterium rectale*) (27, 28). To understand the net metabolism of polyfructans and amino acids by gut microbiota (including the cross-feeding phenomenon), the potential of dominant bacterial groups (pure cultures) to metabolize these compounds needs to be elucidated first.

According to our working hypothesis, the presence, amount, and composition of amino acids should affect fructan utilization by *B. thetaiotaomicron* and alter the profile of produced short-chain fatty acids. To verify this hypothesis, the growth of *B. thetaiotaomicron* in a defined medium was monitored in an isothermal microcalorimeter (IMC) by on-line registration of the heat flow proportional to the growth rate of the bacteria (29). Though IMC has been applied only modestly in microbiology, it is perfectly suitable for anaerobic cultures. The sensitivity of this method – 10^4^ to 10^5^ active bacterial cells – is about 10 times higher than that of other growth detection methods, such as the recording of optical density. Additional advantages of IMC include small working volumes (1–4 mL), applicability for turbid, solid (and anaerobic) environments (30), reduced costs for medium components (including expensive substrates), and increased throughput compared to advanced fermentations. The most important reason for using IMC in this study was the possibility of gas monitoring, which is not possible in the case of microplate screening experiments. On the basis of obtained cultivation data, we aimed to build a quantitative metabolic flux model. Levansucrase Lsc3 of the *Pseudomonas syringae* pv. tomato was used in this study for levan and FOS synthesis. This highly stable and catalytically efficient enzyme produces levan and FOS from sucrose, raffinose, and a cheap sucrose-rich waste product: molasses (12, 31, 32). A feasible method for the synthesis, separation, and purification of levan and FOS is also reported here.

## Materials and Methods

### Bacterial strain and cultivation conditions

The strain *B. thetaiotaomicron* DSM 2079 (Braunschweig, Germany) was used in this study. The bacteria were grown in Fastidious Anaerobic Broth (FAB, LabM, UK) anaerobically (AnaeroGen™, Gas Pack System, Oxoid Inc., UK) at 37°C and preserved at −80°C using Microorganism Preservation System (Technical Service Consultants Ltd., UK). In all experiments the media were inoculated with a cell suspension [inoculation rate about 5–6 log(cfu/mL)] in sterile physiological saline with 0.05% Cys-HCl, prepared from a fresh agar culture (Fastidious Anaerobic Agar, LabM, UK).

### Culture media and substrates

In order to analyze consumption (concentrations) of amino acids and fructans by *B. thetaiotamicron*, the strain was grown in two different phosphate-buffered defined media with the main difference in amino acid content: medium 20 (M20): 20 amino acids, modified from Lahtvee et al. (33), Medium 2 (M2): Cys-HCl and His, based on the Martens et al. (34), also referred as amino acid-restricted medium. The base medium for M20 contained l-amino acids (g/L): Ala 0.044, Arg 0.023, Asn 0.038, Asp 0.038, Glu 0.036, Gln 0.018, Gly 0.032, His 0.027, Ile 0.060, Leu 0.120, Lys-HCl 0.080, Met 0.023, Phe 0.050, Pro 0.041, Ser 0.095, Thr 0.041, Trp 0.009, Val 0.060, Tyr 0.015; mineral salts (mg/L): MgSO_4_ × 7H_2_O 36, FeSO_4_ × 7H_2_O 0.1, CaCl_2_ 9, MnSO_4_ × H_2_O 3, ZnSO_4_ × 7H_2_O 1, CoSO_4_ × 7H_2_O 1, CuSO_4_ × 5H_2_O 1, MgCl_2_ 2, (NH_4_)_6_Mo_7_O_24_ × 4H_2_O 1, NaCl 527, (NH_4_)Cl 400; vitamins (mg/L): biotin 0.25, Ca-pantothenate 0.25, folic acid 0.25, nicotinamide (Niacin) 0.25, pyridoxine-HCl 0.5, B_12_ 0.2, riboflavin 0.25, thiamine-HCl 0.25; 0.5 mL/L Tween 80 and 10 mL/L hemin-vitamin K_1_ solution (hemin – 5 mg/L and vitamin K_1_ 0.5 mg/L in final medium). The base medium for M2 consisted (g/L): His 0.006, MgCl_2_ 0.002, FeSO_4_ × 7H_2_O 0.0001, CaCl_2_ 1, (NH_4_)_2_SO_4_ 0.225, NaCl 0.175; cyanocobalamin (vitamin B_12_) 0.0002 and 10 mL/L of hemin-vitamin K_1_ solution (see above). Both media were prepared in 0.05 M potassium phosphate buffer made of 1 M solutions (mL/L): K_2_HPO_4_ – 35.85 and KH_2_PO_4_ – 14.15. Before the inoculation both media were supplemented with freshly prepared and filter-sterilized Cys-HCl solution (final concentration 0.60 g/L) as a reducing agent and source of sulfur. Solution of autoclaved sodium thioglycolate (0.5 g/L in the final medium, Sigma-Aldrich, Germany) was added to both medium as a reducing agent. The pH of the medium was adjusted to 7.0–7.2 if needed (by adding 1 M NaOH), supplemented with carbohydrate and filter-sterilized (0.2 μm, FP 30/0.2 CA-S, Whatman, Germany). Redox-potential of the culture media at the inoculation, measured by redox electrode (Mettler Toledo, Switzerland) was between −100 to −200 mV.

The following fructans were added one by one to the base medium at concentration of 5 g/L: long-chain inulin HP (DP_ave_ ~23, 0.01% of glc, fru, sucr) (Orafti, Belgium), inulin type fructooligosaccharides (FOS_inu_) (DP_ave_ < 10; glc, fru, sucr ~12%) (Orafti), Lsc3 levan (DP_ave_ ~50, glc, fru, sucr ~4–6%) (University of Tartu, Estonia), yeast treated and concentrated FOS mixture (FOS_mix_, DP 3–8, Table 1) (University of Tartu, Estonia). Differently from other substrates, the FOS_mix_ was liquid and it was added to obtain 5 g/L of FOS in the medium.

**Table 1:** **Sugar composition of the fructooligosaccharide mixture (FOS_mix_) synthesized from sucrose by the Lsc3 and treated with invertase-negative *S. cerevisiae***.

Component	Concentration (g/L)	
	Before yeast treatment	After yeast treatment
Fructose	75.4 ± 16.2	0.1 ± 0.0
Glucose	255.0 ± 19.9	10.6 ± 0.8
Sucrose (DP 2)	44.1 ± 5.9	39.9 ± 3.0
Blastose (DP 2)^a^	9.8 ± 1.1	11.8 ± 2.0
1-kestose (DP 3)^a^	37.0 ± 4.1	42.4 ± 2.8
6-kestose (DP 3)^a^	9.2 ± 1.3	11.3 ± 0.6
Nystose (DP 4)	28.4 ± 1.7	32.1 ± 1.9
Fructosyl nystose (DP 5)	19.7 ± 1.0	24.7 ± 2.1
DP 6	7.0 ± 0.5	11.3 ± 2.2
DP 7	3.6 ± 0.1	3.8 ± 1.5
DP 8	ND	2.1 ± 0.6
Total FOS (DP 3–8)	100 ± 4.4	116.3 ± 12.3
Total sugar (DP 1–8)	481.8 ± 43.2	181.7 ± 12.1

*Sugars were detected and quantified by UPLC. The presented values are average of three measurements with SD*.

*ND, not detected*.

*^a^Verified by MALDI TOF MS and NMR*.

For reference carbon sources, fructose and sucrose (both added at 5 g/L) were used as positive controls and the basal medium without any saccharides added served as a negative control. The sterilized culture media were kept overnight in an anaerobic jar before the inoculation. Inoculation was performed in oxygen-reduced environment flushed with nitrogen gas in a Captair Pyramid Glove Bag (Erlab, Germany).

### Enzymatic synthesis and further treatment of levan and FOS mixture

Levan and FOS_mix_ were synthesized from sucrose using the levansucrase Lsc3 of *P. syringae* pv. tomato (UniProt Q88BN6; ENA HE985190) with very high-catalytic activity and stability (12, 32, 35). Reaction was conducted in 1 L for 20 h at 23°C in 25 mM Na-phosphate buffer (pH 6) containing ~3 U/mL (~7 μg/mL) of purified and filter-sterilized Lsc3 protein and 1.2 M sucrose as a substrate under sterile conditions. The enzyme was inactivated by heating the mixture at 96°C during 10 min and levan was precipitated as in Bekers et al. (10). For that, three volumes of chilled (−20°C) 96% ethanol were added to the reaction mixture and the pH was raised to ~12 with NaOH to aid the sedimentation of underivatized polyfructan. The mixture was kept for 24 h at 4°C and then the precipitate was washed three times with 70% ethanol and freeze-dried in a VirTis freeze dryer (SP Scientific, PA, USA) for 48 h at condenser temperature of 85°C and vacuum of 25 μbar. In every 12 h, the chunks of levan were crumbled. The final humidity of the preparation was between 10 and 15%. The yield of levan was ~80–100 g/L of reaction mixture (up to 13 ± 2.0 g of levan per mg of the catalyst).

The FOS produced by the levansucrase were obtained from the supernatant resulting from levan precipitation. Using a rotary evaporator (Hei-Vap Advantage, Heidolph Instruments, Germany) at 40°C and vacuum of 10–15 mbar, ethanol was removed from the supernatant and the FOS became about five times concentrated. To largely reduce the content of glucose and fructose, fermentation of concentrated supernatant (FOS mixture) with invertase-negative *Saccharomyces cerevisiae* (strain Y02321; EUROSCARF collection) was conducted as described in Yoon et al. (36). Invertase-negative strain of *S. cerevisiae* consumes glucose and fructose from the FOS mixture, whereas not acting on FOS. The yeast was grown in 500 mL YPD medium (g/L: yeast extract 10; peptone 20; glucose 20) at 30°C on a shaker (180 rpm/min) for 20 h. The final OD_600_ of the culture was ~10. The yeast biomass was harvested by centrifugation at 1521 × *g* for 10 min, washed once with sterile mQ water, suspended in the tenfold diluted FOS solution (see above) and incubated for 34 h under static conditions at 30°C. After the incubation, yeast cells were removed by centrifugation, the supernatant was neutralized with 1 M NaOH (to pH 6–7) and further concentrated by vacuum evaporation as described above. The content of saccharides and organic acids in the FOS mixture was determined by HPLC as described below.

The treatment of the FOS mixture with an invertase-negative mutant of *S. cerevisiae* largely decreased the amount of fructose and glucose in it (from about 330 to 10 g/L) while FOS and sucrose were not consumed by the yeast (data not shown). The resulting product, FOS_mix_, contained FOS with DP of 3–8 among which the shorter oligofructans (kestose and nystose) were most abundant (see Table 1). Lysis of yeast cells and formation of unfavorable by-products during the incubation were not observed. Release of mannans from the yeast cells was not observed by HPLC analysis or it remained below the detection limit (data not shown).

### Isothermal microcalorimetry and incubation conditions

The culture ampoules with total volume of 3.3 mL were filled with 2 mL of the inoculated culture, closed hermetically, and incubated at 37°C. The heat flow (P, μW) was automatically recorded in a 24-channel IMC TAM III (TA Instruments, DE, USA) as described by Kabanova et al. (30). Two ampoules from each combination, prepared the same way, were incubated at 37°C for sampling after 24 and 48 h of incubation for HPLC and amino acid analyses. The incubation time was either 72 (first experiment) or 168 h (second experiment). Total heat accumulated (Q, J) was calculated by integration of heat flow which corresponds to biomass amount in the ampoule.

### Analyses

Samples of the culture from the beginning (0 h), 24 h (in some experiments), 48 h and the end of the experiments were analyzed for sugars, organic acids, ethanol, amino acids, amines, poly- and oligofructans, pH, gas composition, and optical density. Biomass concentration was calculated from the power-time curves taking into account heat accumulated (Q) and heat generation coefficient 20 kJ/gDW of biomass produced (37). The biomass yield Y_XS_ (gDW/g-substrate) was defined as the biomass amount produced per carbohydrates consumed.

pH of the culture media and supernatants was measured by pH-meter (Mettler Toledo, MP125, Swizerland), electrode InLab Pro.

Total levan content in culture broth was determined by hydrolysis and anthrone assay as follows. Briefly, samples were diluted and treated with 0.6 M HCl (final concentration) at 70°C for 2 h. Anthrone assay on hydrolyzed samples was performed according to Summerfield et al. (38), except for the anthrone reactive which was prepared as follows: 100 mg anthrone and 2.5 mL absolute ethanol were dissolved in 50 mL 75% (vol%) sulfuric acid in water. The absorbance of samples was measured at 620 nm with a spectrophotometer (Helios Gamma, Thermo Electron Corporation, UK). Standards containing 5–100 mg/L fructose were analyzed with each run. All measurements were performed in triplicate.

Glucose, fructose, sucrose, and FOS with DP up to 18 were quantified from the reaction mixtures as described in Mardo et al. (35, 39). Briefly, chromatography was performed on an Alltech Prevail Carbohydrate ES column (Grace, IL, USA) using Acquity UPLC system (Waters, MA, USA) coupled with evaporative light scattering (ELS) detector. The mobile phase consisted of LC grade water and acetonitrile at flow rate of 0.6 mL/min.

The concentrations of organic acids (succinate, lactate, formate, acetate, propionate, iso-butyrate, butyrate, iso-valerate, and valerate), glycerol, and ethanol in the culture media were analyzed by liquid chromatography (Alliance 2795 system, Waters, MA, USA), using a BioRad HPX-87H column (CA, USA) with isocratic elution of 0.005 M H_2_SO_4_ at a flow rate of 0.6 mL/min and at 35°C. UV (210 nm; model 2487; Waters) and refractive index (RI) (model 2414; Waters) detectors were used for quantification of the substances. Detection limit for the analytical method was 0.1 mM. Samples from culture media were centrifuged (14,000 g, 4 min): supernatants were stored at −20°C until analysis. Before analysis, the samples were thawed, precipitated with 10% sulfosalicylic acid solution in water (1:0.25) and centrifuged. All analyses were performed in duplicate.

Analysis of d- and l-lactate isomers from the culture broth was carried out according to the manufacturer’s instructions (Boehringer Mannheim, R-Biopharm, Germany).

Concentrations of amino acids and amines were determined with an amino acid analyzer (UPLC; Waters, CT, USA) according to the manufacturer’s instructions. Empower software (Waters, USA) was used for the processing of HPLC and UPLC data. Detection limit of the method was 0.01 mM. All standard substrates were of analytical grade. Amino acid analyses were performed in duplicate.

Gas composition (H_2_, CO_2_, H_2_S, CH_4_, and N_2_) of the head of the ampoules was analyzed after 24, 48, and 168 h of incubation using the Agilent 490 Micro GC Biogas Analyzer (Agilent Technologies Ltd., USA) connected to the thermal conductivity detector. Two columns used were CP-Molsieve 5A (for hydrogen, oxygen, nitrogen, and methane) and CP-PoraPLOT U (CO_2_, H_2_S, and propane). The analysis conditions were: sample line temperature 110°C, pressure 200 kPa, and column temperature 80°C. Pure gas standards were used for quantification of individual gases in the sample. It was assumed that nitrogen content was constant in the ampoule during the experiments; therefore it was used to find the amount of gas produced. Between each sample injection, the syringe and the system were flushed with nitrogen gas. The results from the two media were subjected to statistical testing using independent samples *t*-test assuming equal variances and considering both sides of the distribution (one-tailed distribution). Samples were considered as significantly different if *p*-value was below 0.05.

### Metabolic flux analysis

A simplified metabolic network was constructed based on metabolic reaction information of *B. thetaiotaomicron* VPI-5482 from SEED database (theseed.org). The central pathways (glycolysis, pentose phosphate cycle, and pyruvate metabolism), amino acid metabolism and biomonomer synthetic fluxes were taken into account to calculate the reactions (Data Sheet S1 in Supplementary Material, Table S1 in Data Sheet in Supplementary Material – list of reactions used for flux calculation, Table S2 in Data Sheet in Supplementary Material – list of metabolites involved in the reactions, Table S3 in Data Sheet in Supplementary Material – biomass composition used for flux calculations, Table S4 in Data Sheet in Supplementary Material – raw data measured, Table S5 in Data Sheet in Supplementary Material – flux values of measured substrates and products, Table S6 in Data Sheet in Supplementary Material – calculated flux values). Change of Cys was not considered as consumption in the metabolic model, since most probably significant part of it was degraded in reaction with oxygen and only some cysteine depleted from the medium was consumed by bacteria [see Carbonero et al. (40)].

The metabolic network contains 79 independent fluxes (measured in this study or taken from literature) and 240 dependent (calculated) fluxes. The calculated fluxes comprise measured consumption of 20 amino acids, simple sugars (glucose, sucrose, or fructose), and oligosaccharides, formation of fermentation by-products (acetate, butyrate, ethanol, formate, glycerol, lactate, propionate, succinate), formation of amino acid degradation products (iso-butyrate, iso-valerate, valerate, cadaverine, histamine, putrescine, tryptamine, tyramine), gas production (CO_2_, H_2_, H_2_S), and production of biomass components (peptidoglycan, lipids, amino acids, and nucleotides in total 35 components taken from biomass of *Escherichia coli* at specific growth rate 0.35 h^−1^ (41) as no full data about any *Bacteroides* stain is available). Four independent fluxes are: balanced fluxes of carbon, nitrogen, ATP, and NAD(P)H. Measured concentrations of substrates and products (mM) were converted to productions/consumptions per biomass dry weight (mmol/gDW) assuming that an average biomass conversion factor is 20 kJ/gDW (37). Metabolic network was fully determined and spreadsheet was used to perform the calculations.

## Results

### Growth of *Bacteroides thetaiotaomicron* in defined media

**Figure 1:**
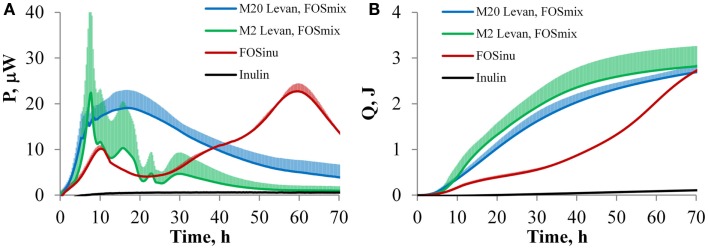
**The curves of power (A) and accumulated heat (B) with SD during the growth of *B. thetaiotaomicron* DSM 2079 in the defined media M2 and M20 (2 and 20 amino acid added, respectively)**. Blue line: two replicates with each substrate, i.e., fructose, sucrose, levan, and FOS_mix_ in the M20 medium are grouped together. Green line: two replicates with each substrate, i.e., fructose, sucrose, levan, and FOS_mix_ in the M20 medium are grouped together. Red line: two replicates with FOS_inu_ in the M20 medium.

**Figure 2:**
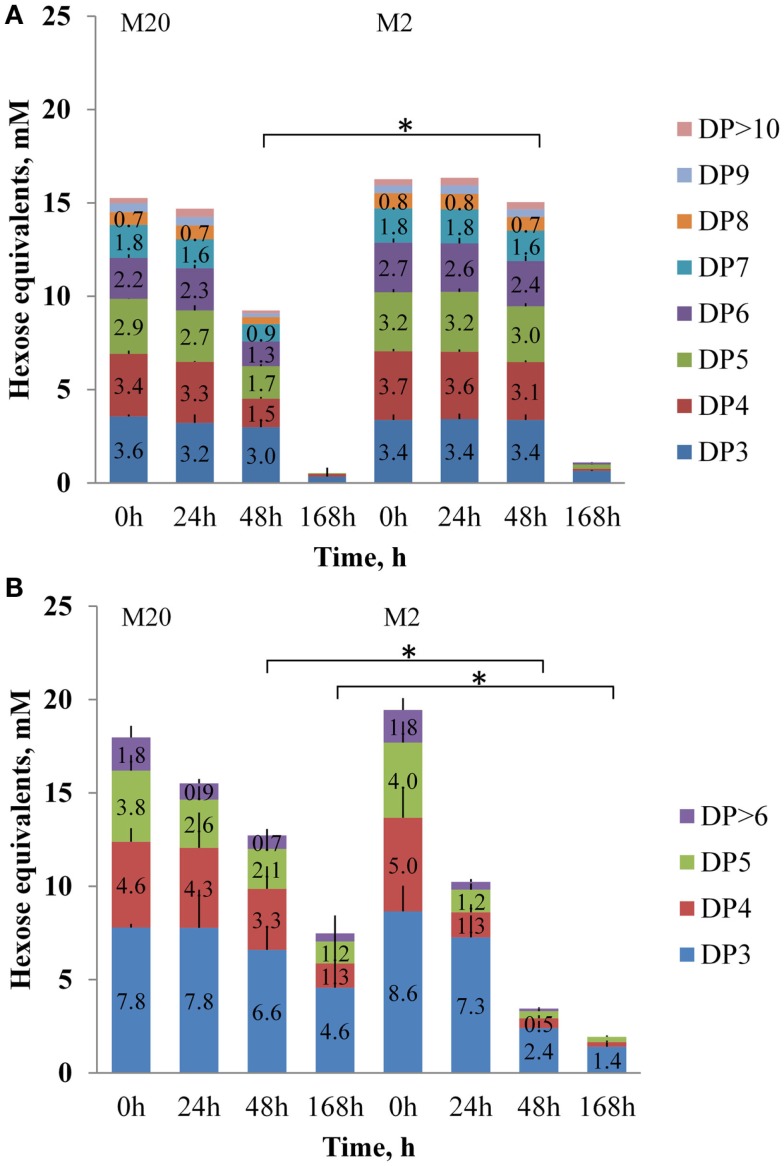
**Concentrations of fructooligosaccharides FOS_inu_ (A) and FOS_mix_ (B) of different degree of polymerization (DP, as hexose equivalents, mmol/L) at growth of *B. thetaiotaomicron* at 37°C (0, 24, 48, and 168 h) in the defined media M20 and M2**. Error bars indicate the absolute deviation of two parallel experiments. Asterisk indicates *p*-value below 0.05.

Data on heat generation for on-line growth analysis and oligosaccharide fermentation by *B. thetaiotaomicron* were used for the first time. The growth of *B. thetaiotaomicron* DSM 2079 was observed in defined media containing either twenty (M20 medium) or two (M2 medium) amino acids and mono-, oligo-, or polyfructans, but not in the absence of carbohydrates. The characteristic power-time (A) and heat accumulation (B) curves of *B. thetaiotaomicron* grown in defined media are presented in Figure 1. The change of the heat flow (P, μW) correlates with the growth rate, and the accumulation of heat (Q, J) is related to the biomass amount. More clearly differentiated heat peaks were produced in the M2 medium (Figure 1A). The metabolism of fructose and sucrose by *B. thetaiotaomicron* resulted in power-time curves similar to that of the polyfructan levan (data not shown). It can be seen that the majority of biomass was formed before the 48^th^ hour (Figure 1B). FOS_inu_ was a clear exception among the fructans used, providing the slowest and most protracted growth. With both amino acid compositions, two clearly separable heat peaks were observed during growth on FOS_inu_, while the shortage of amino acids, though prolonging the lag-phase, had no influence on the overall pattern of the power-time curves and final biomass yields (only the M2 medium is presented in Figure 1A). In the M2 medium, the lag phase was longer and active growth of *B. thetaiotaomicron* occurred mostly after 48^th^ hours of incubation (not shown in Figure 1). That is in accordance with the changes of substrate concentration and product formation (Figures 2A,B; Table 2). It should be noted that the sampling points are not distributed evenly – time between 24 and 48 h is remarkably shorter (24 h) compared to that from 48 up to 168 h (96 h).

**Table 2:** **Consumption of amino acids and formation of products (mmol/gDW) in defined media M20 and M2 (containing 20 and 2 amino acids, respectively) during the growth of *B. thetaiotaomicron***.

	M20 medium			M2 medium		
	0–24 h	24–48 h	48–168 h	0–24 h	24–48 h	48–168 h
	Ave ± SD	Ave ± SD	Ave ± SD	Ave ± SD	Ave ± SD	Ave ± SD
Sum-Carb	228 ± 111	209 ± 43	132 ± 33	180 ± 48	203 ± 106	148 ± 37
Sum-AA	-0.0 ± 1.6	8.0 ± 3.2	12.4 ± 10.9	0 ± 0	-7.6 ± 4.5	14.4 ± 16.8
Ala	0 ± 0	-0.6 ± 0.9	-1.3 ± 1.5	ND	-2.0 ± 0.5	0.2 ± 1.7
Asp	0.0 ± 1.2	2.8 ± 0.6	5.3 ± 4.2	ND	-0.1 ± 0.2	0.9 ± 1.4
Gln	-0.1 ± 0.3	0.5 ± 0.3	2.5 ± 1.4	ND	0.0 ± 0.1	0.2 ± 0.4
Glu	0 ± 0	-0.7 ± 0.8	3.5 ± 3.1	ND	-1.7 ± 0.9	7.9 ± 4.2
Gly	0 ± 0	0.2 ± 0.5	0 ± 0	ND	-0.5 ± 0.4	0.7 ± 1.5
Ser	0 ± 0	2.7 ± 1.0	1.3 ± 1.8	ND	-0.2 ± 0.1	0.2 ± 0.3
Thr	0 ± 0	1.2 ± 0.4	0.5 ± 1.0	ND	-0.1 ± 0.0	0.0 ± 0.2
H_2_	6.2 ± 5.5	-0.5 ± 1.1	0.9 ± 2.8	14.3 ± 3.9	2.7 ± 2.7	-5.4 ± 7.9
CO_2_	19 ± 13	15 ± 6.8	2.9 ± 12.3	16.6 ± 4.8	12.3 ± 8.2	-11 ± 29
Ace	83 ± 46	52 ± 4.5	30 ± 38	57 ± 38	92 ± 55	15 ± 30
Lact	56 ± 34	82 ± 32	17 ± 35	0.08 ± 0.1	64 ± 38	53 ± 51
Prop	57 ± 39	63 ± 22	66 ± 54	18 ± 12	90 ± 31	128 ± 86
Succ	45 ± 31	22 ± 14	88 ± 94	36 ± 26	68 ± 37	0 ± 0

*Data on levan, FOS_mix_, sucrose, and fructose were grouped together and average values were calculated for both media compositions. Sum-AA, the sum of consumption of all amino acids. Sum-Carb, the sum of consumption of carbohydrates (in hexose equivalents). Negative values for amino acids and gases indicate the production. ND – not determined*.

The obtained power-time curves (except for FOS_inu_) can be analyzed in phases: the first and rapid heat generation phase (up to 24 h of growth), and the second slow heat accumulation phase (between 24 and 48 h of growth), followed by a gradual ceasing thereafter (Figure 1B). Considering the similarities between the power-time curves and product formation patterns, the results on levan, FOS_mix_, sucrose and fructose were grouped together. Thus, the growth of *B. thetaiotaomicron* in two media with different amino acid compositions (M20 and M2) was compared for this substrate group (Figure 1; Table 2).

The average growth rates (μ, 1/h) during the first (fast) growth phase in the M20 medium were 1.02 ± 0.11 h^−1^ and in the M2 medium 0.82 ± 0.18, and generally the fructan type had no effect on them. The fastest growth of *B. thetaiotaomicron* was observed with levan in the M2 medium (data not shown). By 48 h of incubation, 75% of the levan had been consumed and 90% of the final biomass had formed. The average biomass yields per substrate consumed (Y_XS_) on all substrates and both amino acid compositions were 0.04 ± 0.01 gDW/g-hexoses.

### Consumption of substrates and synthesis of products

**Figure 3:**
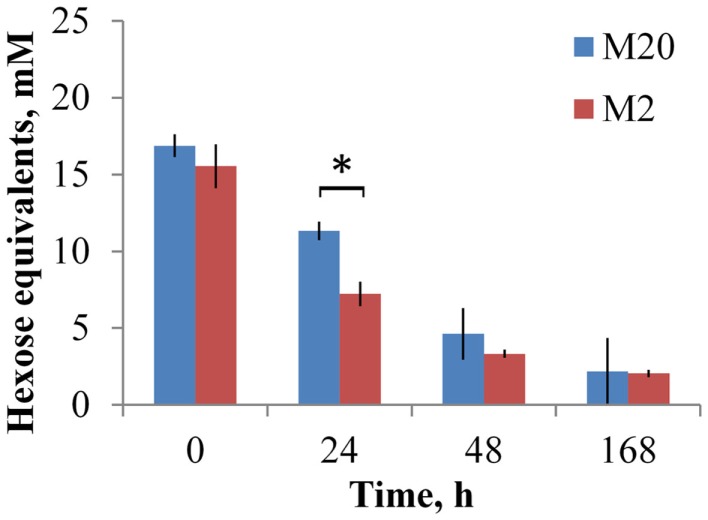
**Concentration of levan at growth of *B. thetaiotaomicron* at 37°C in the M20 medium containing 20 amino acids and M2 medium containing 2 amino acids**. Error bars indicate the absolute deviation of two parallel experiments. Asterisk indicates *p*-value below 0.05.

Analyses of the oligosaccharides revealed that *B. thetaiotaomicron* metabolized oligofructans with various DPs from both FOS preparations. As shown in Figure 2A, *B. thetaiotaomicron* effectively metabolized FOS_inu_ species with chain lengths up to 20 oligomers (fraction marked as > 12 monomers). In the M20 medium, during the first 24 h of growth mostly mono- and disaccharides were metabolized from FOS_inu_ (about half of their total amount; Data Sheet S1 in Supplementary Material), while oligomers with DP over 3 were consumed thereafter in parallel with the largest heat peak. In the M2 medium, the oligosaccharides of FOS_inu_ were consumed only after 48 h of growth. From FOS_mix_, oligosaccharides with DP > 5 (in the M2 medium) and DP > 7 (in the M20 medium) were totally metabolized, while shorter oligosaccharides were only partly metabolized (Figure 2B). In parallel with the consumption of FOS_inu_ and FOS_mix_, the accumulation of fructose was observed, especially in growth in the M2 medium (data not shown). Similar consumption of fructose and oligofructose by *B. thetaiotaomicron* LMG 11262 has been reported by Van der Meulen et al. (42). Compared to the medium M20, increased consumption of levan (69 vs. 84% of the total) and FOS_mix_ (60 vs. 90%) was observed in the M2 medium (Figures 2 and 3), possibly due to additional expenditures on protein synthesis.

The amino acids metabolized most actively by *B. thetaiotaomicron* were Asp, Ser, and Thr, the consumption of which during the first 24 h in the M20 medium was 2.8 ± 0.6, 2.7 ± 1.0, 1.2 ± 0.4 mmol/gDW, respectively (Table 2). The production of Ala, especially in the amino acid deficient medium, was observed on all substrates; Glu was produced up to 48 h and consumed thereafter (Table 2).

**Figure 4:**
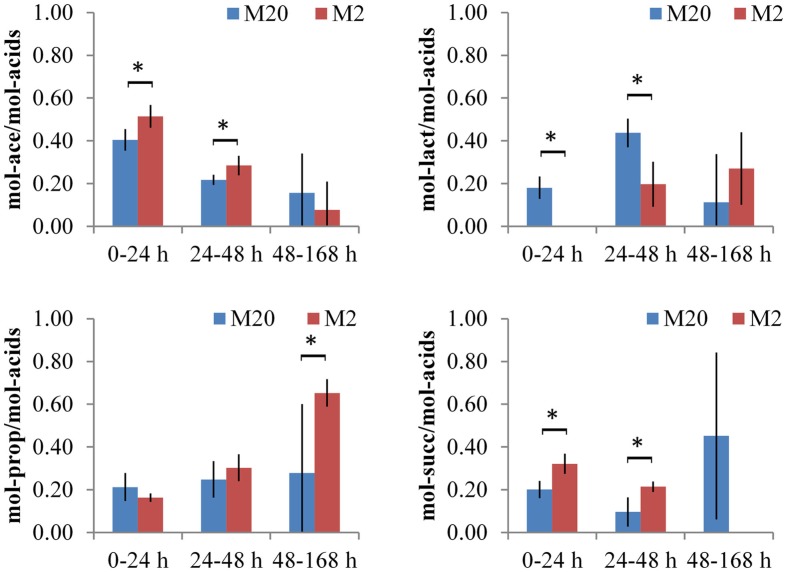
**Production of organic acids by *B. thetaiotaomicron* in defined media, containing 20 or 2 amino acids (M20 and M2), respectively**. Experiments with fructose, sucrose, levan, and FOS_mix_ are grouped together and average values of duplicate experiments are shown with absolute deviations. For each time interval (0–24, 24–48, and 48–168 h) the sum of all acids is normalized to 1 and ratios (expressed as mol/mol) of acetate/total acids (Ace), lactate/total acids (Lact), propionate/total acids (Prop), or succinate/total acids (Succ) are presented. Asterisk indicates *p*-value below 0.05.

The main metabolic products of *B. thetaiotaomicron* in defined media were lactic, acetic, propionic, and succinic acids (in some cases very small amounts (3 mmol/gDW) of formate were produced). As mentioned above, growth data on levan, FOS_mix_, sucrose, and fructose were analyzed as a group on two media with different amino acid contents (M20 and M2). The total amounts of organic acids produced were similar in both media (M20 and M2), although with varying proportions and formation dynamics depending on the amino acid supply (Table 2; Data Sheet S1 in Supplementary Material). In the M20 medium, the acetic acid production dominated during the initial fast growth phase (up to 24 h), followed by increased production of lactic (from 24 to 48 h of growth), propionic, and succinic acids (from 48 to 168 h of growth). The product profile was different in the M2 medium, in which almost no lactic acid was produced during the first 24 h of growth, and no succinic acid was produced after 48 h of growth. Instead, the deficiency in amino acids enhanced the production of propionic acid in the last phase (between 48 and 168 h) (Figure 4).

In parallel with the increase in biomass up to 48 h of growth, enhanced production of acetic acid, H_2_, and CO_2_ occurred to balance the redox potential during acetate production.

The average pH change in all experiments at about 1.5 pH units correlated inversely with heat accumulation (increase in biomass) and was quite similar in all substrates. The final pH at the end of experiment (168 h) was 5.6 ± 0.2 and 5.4 ± 0.3 in the M20 and M2 media, respectively. Slightly lower pH (except for sucrose) was achieved in the M2 medium, which is in accordance with faster and somewhat elevated consumption of fructans (Figures 2 and 3).

## Discussion

### Metabolism of fructans

Whereas plant-derived inulin and related FOS are industrially produced, food-grade levan, and levan-type FOS are still not commercially available. In the current study, levan and FOS were enzymatically prepared at laboratory-scale. The growth of the common gut bacterium *B. thetaiotaomicron* on these substrates was addressed with a focus on the effect of an amino acid supply (20 vs. 2 amino acids) on fructan metabolism. For improved quantification of metabolic fluxes, all experiments were carried out in defined media. The most important growth differences described here are apparently related to the different amino acid composition of the two media, although variations in vitamin and mineral contents may also influence the growth and metabolism of *B. thetaiotaomicron* to some extent (19).

As hypothesized, the growth characteristics of *B. thetaiotaomicron* on fructans varied depending on the amino acid composition of the medium. The multiauxie of heat production can be explained by gradual degradation of complex carbohydrates. However, also in the case of growth on fructose and sucrose as substrates, the detailed carbon balance revealed a gap between substrate consumption and product formation (Data Sheet S1 in Supplementary Material), which may refer to the production of unknown metabolites, such as oligosaccharides, during the initial growth (up to 24 h) and their stepwise degradation at a later stage. According to Sonnenburg et al. (43), glycan composition in the diet of *B. thetaiotaomicron*-colonized mice affected the expression of capsular polysaccharides synthesis loci of the bacteria. However, that may not be valid under *in vitro* conditions. Sucrose is a suitable energy-rich substrate for several bacterial extracellular enzymes to synthesize poly- and oligosaccharides. Inspection of the CAZy database did not disclose sucrose-polymerizing enzymes (levansucrases, inulosucrases, glucansucrases) in *B. thetaiotaomicron*. As bacterial biomass was not analyzed for the presence of specific enzymes nor polysaccharides in this study, this issue has to be clarified in further experiments.

A more constant heat flow (without sharp peaks) at growth in M20 medium may result from the simultaneous metabolism of fructans and amino acids without the need to switch between different carbon and energy sources. The two-phase growth observed in the case of FOS_inu_ (Figure 1) suggests that certain oligofructan species (consumed first) are preferred over others and are consumed only after exhaustion of the preferred fractions. The FOS_inu_ preparation contains FOS fractions with different chain lengths and different composition: (i) the β 2,1 linked oligofructose chains resulting from the cleavage of the main chain of the inulin (FF_n_), and (ii) the oligofructose chains capped with glucose residues originating from the chain-initiating sucrose molecule (GF_n_). Also, the transport and degradation systems of oligofructans can be more efficient than those of monosaccharides, whereas fructose stimulates fructan utilization (17). Accumulation of fructose in the M2 medium that we observed probably reflects the breakdown of longer oligosaccharides. This phenomenon has also been reported by Van der Meulen et al. (42) during growth of *B. thetaiotaomicron* LMG 11262 in a complex medium supplemented with an oligofructose preparation (Raftilose P95, Orafti). However, unlike our experiments, all fructose was consumed from the medium after the exhaustion of oligosaccharides. This may indicate to some differences between the stains, or can be caused by different pH profile or medium constituents.

The inability of *B. thetaiotaomicron* to metabolize inulin is consistent with the data of Sonnenburg et al. (17). Thus, the growth of *B. thetaiotaomicron* DSM-2079 on inulin shown in another study (21) could possibly be explained by the presence of a fermentable sugar in the yeast extract and/or some impurities (monosaccharides and FOS) in the inulin preparation.

The production of lactic acid by *B. thetaiotaomicron* as fermentation product is controversial to the literature data – only few studies have mentioned it (19). We detected the production of d-lactic acid at fermentation of all tested fructans, whereas l-lactic acid was not detected (Figure 4). It is known that d-lactic acid is not absorbed from the human intestine, and can be converted into acetic and butyric acids by colon bacteria with the concurrent generation of CO_2_. Our results showed that extensive consumption of amino acids, especially energetic amino acids Ser, Thr, and Asp (up to 12 mmol/gDW on average, Table 2) stimulated production of d-lactic acid in M20 medium, whereas formation of acetic acid and hydrogen gas was inhibited. In comparison, succinic and acetic acids were the main metabolites of *B. thetaiotaomicron* LMG 11262 produced in a complex medium, while a small amount of propionic acid was produced only from oligofructose (42). In these experiments, however, pH was changed from 5.8 to 6.8, which might have affected the metabolism of the saccharides as well as formation of products.

### Metabolism of amino acids

Although *B. thetaiotaomicron* does not need amino acids for growth, their presence or absence obviously affected the metabolism of fructans. Our results confirmed the ability of *B. thetaiotaomicron* to synthesize the necessary amino acids from inorganic ammonia (ammonium sulfate in M2 medium) as already reported by Varel and Bryant (19). The human colon, the normal environment of *B. thetaiotaomicron*, probably contains very low concentrations of free amino acids due to their efficient absorption from the small intestine (44). However, some amino acids or peptides might be delivered during the breakdown of the host mucin and dead epithelial and microbial cells. Importantly, the amino acids with the highest consumption level: Asp, Ser, and Thr, are major components of the mucin (45) indicating excellent adaptation of *B*. *thetaiotaomicron* to its ecological niche. The calculated consumption of total amino acids per total hexose equivalents (mol/mol) in the case of the M20 medium was about two times higher for FOS_inu_ than for FOS_mix_ and levan (0.18 vs. 0.07 and 0.06 mol/mol, respectively).

**Figure 5:**
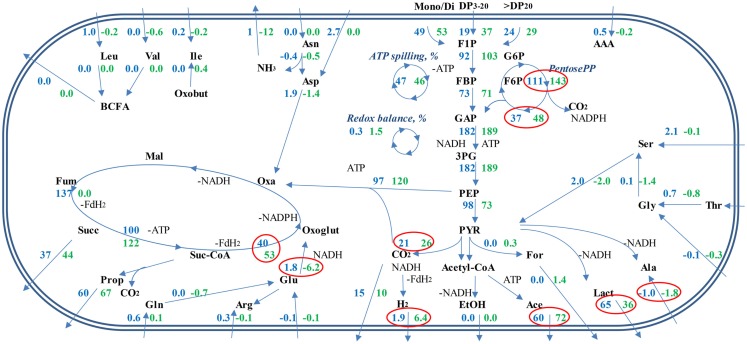
**Proposed metabolic fluxes in *B. thetaiotaomicron* DSM 2079 grown in defined media**. Average combined fluxes on levan, FOS_mix_, sucrose, and fructose in M20 medium (blue numbers), and M2 medium (green numbers). Significantly different fluxes (*p* < 0.05) at growth on M20 and M2 media are surrounded with red circles. Relative ATP spilling (%) shows the amount of ATP from total ATP produced not used for substrate transport, biomonomer synthesis, and polymerization of macromolecules. Relative redox balance (%) illustrates the amount of oxidized redox equivalents from total produced not regenerated through formation of measured fermentation end-products. Mono/Di means the sum of mono- and disaccharides (fructose, glucose, sucrose). DP is the degree of polymerization for oligo- and polysaccharides.

Based on the experimental data, the stoichiometry of carbon and nitrogen metabolism at growth in M20 and M2 media was proposed (Figure 5; Data Sheet S1 in Supplementary Material). A comparison of the production of ATP and the formation of redox equivalents (NADH or reduced ferredoxin) in the production of organic acids (acetic, lactic, propionic, and succinic acids) from phosphoenolpyruvate (PEP) revealed that the production of acetic acid is energetically most efficient as it yields two molecules of ATP, with the production of one equivalent of CO_2_ and one redox equivalent (NADH). The production of lactic, propionic, or succinic acids requires external redox equivalents (1, 2, and 2 per 1 molecule of lactic, propionic, and succinic acid, respectively) while generating only 1 ATP per reaction leading from PEP to organic acid. In comparison, 1 redox equivalent per PEP produced is generated in glycolysis that is ATP-neutral (2 ATP-s produced and 2 used for glucose uptake and phosphorylation). The formation of propionic acid from lactate by *B. thetaiotaomicron* has not been shown (see the KEGG database, http://www.genome.jp/kegg/). A prerequisite for the production of succinic acid through the reductive tricarboxylic acid (TCA) cycle is the availability of carbon dioxide, which can either be formed in the conversion of acetyl-CoA to pyruvate or taken from the extracellular environment.

Concerning the average composition of the bacterial biomass (data on *Escherichia coli* from (41), as no appropriate data on *Bacteroides* spp. is available, Data Sheet S1 in Supplementary Material; Table S3 in Data Sheet in Supplementary Material), it can be calculated that the total consumption of amino acids exceeds the need for the synthesis of the biomass proteins about 2–4 times (5.0 vs. 8.0–12.4, Table 2). That indicates extensive use of amino acids either for energy, for ammonia supply or for balancing the redox potential as (these) amino acids can be easily converted to key intermediates (pyruvate, oxaloacetate) of central metabolic pathways. It is important to note that Ala and Glu were produced in all media (though in three times higher amount in M2 than in M20 medium, Table 2) that can be related to regeneration of NAD^+^. Although total amino acid consumption exceeded by several times the requirements for the synthesis of bacterial proteins, it remained only marginal (12.4 mmol/gDW in the M20 medium) compared to consumption of carbohydrates (107 mmol/gDW). Yet, the effect of amino acids on the profile of organic acids produced was significant, as considerably more propionic acid and less d-lactate was formed under amino acid deficiency (Figure 5). This product shift was obtained by increased fluxes through acetyl-CoA and anaplerotic oxaloacetate synthesis, which was accompanied by increased throughput via the TCA cycle and reduced regeneration of NAD^+^ through lactate synthesis. The role of the reduced regeneration of electron acceptors via lactate synthesis in M2 medium is also partially related to increased fluxes through glutamate dehydrogenase and hydrogen production (by more than three times), which consume NADPH and NADH/FADH_2_, respectively.

In conclusion, we showed that the fructan metabolism of *B. thetaiotaomicron* DSM 2079 depends on the amino acid supply. A surplus of amino acids reduces consumption of longer-chain FOS (DP > 3) and flux through the reductive TCA cycle compared to growth under amino acid deficiency. The metabolism of FOS is related to their composition and structure: considerable differences were disclosed between the metabolism of two FOS preparations explored in the current study. This knowledge should be taken into account when addressing the prebiotic properties of different types of FOS as promoters of production of SCFA and other beneficial metabolites under varied nutritional conditions. A shortage of amino acids was shown to stimulate the production of propionic acid by *B. thetaiotaomicron*. The polyfructan levan showed a great potential for selective modification of gut microbiota, a possibility that will be studied in further experiments.

## Conflict of Interest Statement

The authors declare that the research was conducted in the absence of any commercial or financial relationships that could be construed as a potential conflict of interest.

## Supplementary Material

The Supplementary Material for this article can be found online at http://www.frontiersin.org/Journal/10.3389/fnut.2014.00021/abstract

Data Sheet S1 **Metabolic flux calculations**. Reactions, metabolites, and biomass composition used for metabolic flux analysis (Tables S1, S2, and S3 in Data Sheet in Supplementary Material, respectively), raw data measured in mM and mmol/gDW (Tables S4 and S5 in Data Sheet in Supplementary Material, respectively) and calculated values of intracellular fluxes of all experiments (Table S6 in Data Sheet in Supplementary Material).
